# Ginsenosides Enhanced Apoptosis of Serum-Free Starved A549 Lung Cancer Cells

**DOI:** 10.3390/molecules30183697

**Published:** 2025-09-11

**Authors:** Jiwen Li, Keke Li, Mei Sun, Zhihong Gu, Lei Men, Xiaojie Gong, Zhongyu Li

**Affiliations:** 1 School of Biological Engineering, Dalian Polytechnic University, Dalian 116034, China; ljw131009@163.com (J.L.); sunny201905@yeah.net (M.S.); 2 College of Life Science, Dalian Minzu University, Dalian 116600, China; like905219@163.com (K.L.); 17864226819@163.com (Z.G.); menlei@dlnu.edu.cn (L.M.)

**Keywords:** ginsenosides, lung cancer, serum starvation, apoptosis, proliferation, PI3K/Akt/FoxO signaling pathway

## Abstract

Lung cancer remains a leading cause of cancer-related mortality worldwide, where conventional chemotherapy is often limited by severe side effects and drug resistance. Ginsenosides, the primary bioactive triterpenoid saponins isolated from the root of Panax ginseng C. A. Mey, have demonstrated potential in combating non-small-cell lung cancer (NSCLC). However, their efficacy under nutrient-deficient conditions remains unclear. This study aimed to investigate the effects of ginsenosides on the growth and death of lung cancer cells under low-nutrient conditions and to explore the underlying mechanisms. A549 cells were divided into two groups: one cultured in 10% serum and another under serum-free conditions, followed by treatment with ginsenosides CK, Rh2(S), and Rg3(S) for 24 h. Cell proliferation and apoptosis were evaluated using a CCK-8 assay, Calcein/PI fluorescence staining, Hoechst 33258 staining, and flow cytometry. Potential targets and signaling pathways of ginsenosides were predicted using network pharmacology and bioinformatics analyses. The mRNA expression of key genes was measured by qRT-PCR, and mitochondrial membrane potential was assessed using JC-1 staining. The results showed that ginsenosides induced dose-dependent apoptosis in serum-starved A549 cells. Bioinformatics analysis suggested the involvement of the PI3K/Akt/FoxO signaling pathway, which was supported by decreased Akt mRNA levels and increased FoxO mRNA expression. Furthermore, mRNA levels of Bim, Caspase-3, Caspase-8, and Caspase-9 were significantly upregulated, accompanied by a loss of mitochondrial membrane potential. These findings indicate that under serum deprivation, ginsenosides enhance apoptosis in A549 cells, likely through the regulation of the PI3K/Akt/FoxO pathway.

## 1. Introduction

Lung cancer represents a leading cause of cancer-related mortality worldwide, characterized by high mortality rates and poor prognoses [1]. It is categorized into two main types: small-cell lung cancer and non-small-cell lung cancer (NSCLC), with the latter constituting the majority, accounting for approximately 85% to 90% of all lung cancers [2]. Currently, chemotherapy remains the cornerstone of the clinical management of lung cancer. However, it is regrettable that commonly used chemotherapy drugs, such as cisplatin, exhibit serious side effects due to their lack of selectivity towards tumor cells [3]. Additionally, as treatment progresses, chemotherapy drugs frequently induce the development of drug resistance, significantly compromising their efficacy [4]. Hence, there is an urgent imperative to explore novel anti-lung cancer drugs capable of acting through novel mechanisms to overcome these challenges. The discovery of novel anti-lung cancer drugs from natural products is regarded as a promising approach [5].

Ginseng (Panax ginseng C. A. Mey) has been utilized for medicinal purposes for centuries in numerous Asian nations, including China, Japan, and South Korea [6]. The plant’s therapeutic properties are meticulously documented in *Shen Nong’s Herbal Classic*, one of the earliest pharmacological texts in China, highlighting its efficacy in nourishing the internal organs, sedating the nervous system, enhancing cognitive abilities, and prolonging life expectancy [7]. Contemporary medical research has substantiated the plant’s pharmacological activities, including anti-tumorigenic, antioxidative, and anti-inflammatory effects, which are recognized in the Chinese pharmacopoeia [8,9]. Of particular interest are the anti-tumor properties of specific ginsenosides, such as Rg3(S), Rh2(S), and CK, which have been validated in recent experimental and clinical research [10]. Notably, the ginsenoside Rg3 has been developed into an anti-tumor therapeutic agent, “ShenYi Capsule,” and has been incorporated into the National Comprehensive Cancer Network guidelines (2016) [11]. The induction of cancer cell apoptosis is considered one of the primary underlying mechanisms by which ginsenosides exert their anti-cancer effects. The ginsenoside Rg3 is able to trigger cell death in lung cancer cells by targeting the neurogenic locus notch homolog protein (Notch)/hairy and enhancer of split 1 (HES1) pathway and suppressing growth in NCI-H1650, H520, and H1963 cells [12]. The ginsenoside Rh2 stimulates the c-Jun N-terminal kinase (JNK)/mitogen-activated protein kinase (MAPK) signaling pathway, impacting the levels of the cell cycle proteins D1 and cyclin-dependent kinase 4 (CDK4), leading to cell cycle arrest at the G1 phase and subsequent cell death in A549 cells [13]. The ginsenoside CK significantly boosts the activity and expression of cisplatin-induced tumor protein 53 (p53) in H460 and A549 cells, resulting in combined cell death in synergy with cisplatin [14]. Previous research has demonstrated that these ginsenosides can trigger cell death to various extents in A549 cells [15].

Insufficient blood supply in solid tumors leads to changes in the intra-tumoral microenvironment, affecting the aggregation of essential nutrients, oxygen, and metabolic byproducts crucial for tumor cell survival [16,17]. Specifically, malnutrition incites cellular events within the depths of tumors due to incomplete vascular perfusion [18]. Particularly challenging is the nutritional deficit faced by lung carcinoma cells, given their elevated proliferative index, particularly evident during initial tumorigenesis [19]. In vitro, serum provides vital nutrients like growth factors, hormones, and amino acids [20]. Thus, serum starvation is a common methodology to mimic in vivo nutrient deprivation in cancer cell investigations [21]. Additionally, serum inclusion in signal transduction studies may compromise experimental accuracy [22]. Therefore, serum-free culturing conditions facilitate the more-precise assessment of drug impact on tumor cells, encompassing parameters such as proliferation, apoptosis, and metastatic potential, thus aiding in the screening and evaluation of prospective anti-neoplastic agents or therapeutic modalities [23,24]. For instance, cisplatin increases sensitivity in lung and colorectal carcinoma cells under serum deprivation conditions by enhancing the activation of the ataxia telangiectasia mutated (ATM)/checkpoint kinase 2 (Chk2)/P53 signaling axis relative to normal culture conditions [25]. Similarly, extracts of Radix Rehmanniae stimulate the phosphorylation of p38 mitogen-activated protein kinase (p38) and JNK in colorectal carcinoma cells under serum deprivation, leading to cell cycle arrest and apoptosis [26]. Notably, extracts from Calotropis gigantea stem bark inhibit colorectal carcinoma cell proliferation under serum deprivation [27]. Although ginsenosides have been promoted for their anti-neoplastic efficacy, elucidating their anti-tumor effects and mechanistic underpinnings under conditions of nutritional deprivation warrants further investigation.

In this study, we employed in vitro serum starvation to simulate the nutrient-deprived microenvironment of cancer cells in vivo. We investigated the effects of ginsenosides on the proliferation and apoptosis of NSCLC A549 cells. Concurrently, we employed network pharmacology and bioinformatics approaches to predict potential mechanisms of action and validated signaling pathways associated with nutrient metabolism. Our findings contribute to a deeper understanding of the anti-tumor mechanisms of ginsenosides, providing novel theoretical insights and leads for their development as clinical drugs.

## 2. Results and Discussion

### 2.1. Ginsenoside Inhibits the Proliferation of A549 Cells Under Serum Starvation

**Figure 1:**
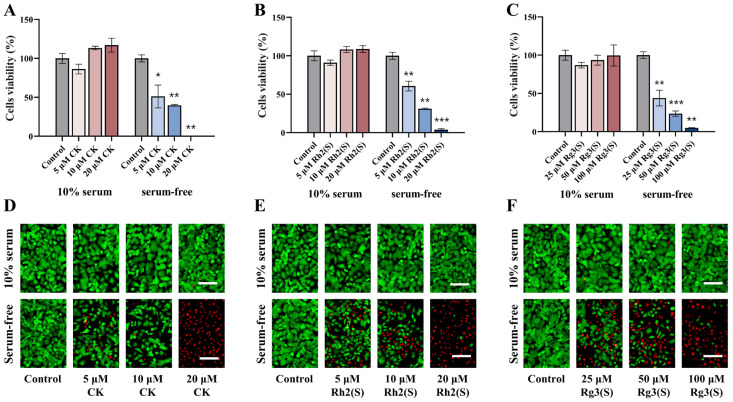
Effect of ginsenosides on the proliferation of A549 cells under different culture conditions. Quantitative assay of cell viability of A549 cells treated with ginsenosides CK (**A**), Rh2(S) (**B**), and Rg3(S) (**C**) under different culture conditions. Fluorescent images of ginsenosides CK (**D**), Rh2(S) (**E**), and Rg3(S) (**F**) acting on A549 cells without and with serum. Scale bar: 100 μm. Error bars represent mean ± SD; *n* = 3. (* *p* < 0.05, ** *p* < 0.01, and *** *p* < 0.001 vs. the control group).

**Figure 2:**
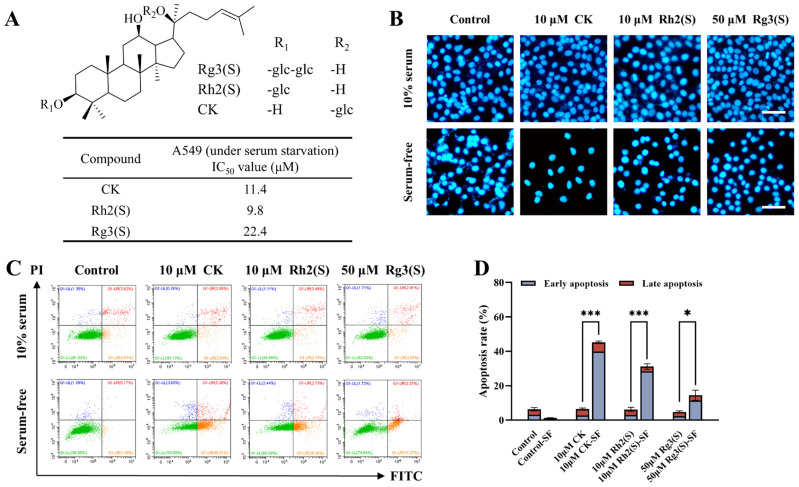
Effect of ginsenosides on the apoptosis of A549 cells under different culture conditions. (**A**) Chemical structures and half-maximal inhibitory concentrations (IC_50_) of ginsenosides. (**B**) Morphological assessment of apoptosis by Hoechst 33258 staining. Scale bar: 50 μm. (**C**) Dot plots showing apoptosis through the flow cytometric analysis of A549 cells using dual annexin V/FITC-PI staining. (**D**) Comparison of apoptosis levels across various experimental conditions. Apoptosis ratio was calculated by combining early apoptosis and late apoptosis percentages. Error bars represent mean ± SD; *n* = 3. (* *p* < 0.05, *** *p* < 0.001).

The experiment comprised two groups: one exposed to 10% serum and another to serum-free conditions, with each group treated with ginsenosides CK, Rh2(S), and Rg3(S) for 24 h on A549 cells. Cell viability was evaluated using the cell counting kit-8 (CCK8) assay, and the results are depicted in Figure 1A–C. Corresponding fluorescent images representing cell viability are provided in Figure 1D–F, with green fluorescence indicating viable cells and red fluorescence indicating dead cells. As depicted, under conditions of 10% serum culture, no significant difference in cell viability was observed between the treatment and control groups. However, the induction of serum starvation in A549 cells using a serum-free culture medium and subsequent treatment with varying concentrations of ginsenosides resulted in varying degrees of cell death. Specifically, after 24 h of treatment with low concentrations of ginsenosides CK, Rh2(S), and Rg3(S), the viability of A549 cells was measured at 51.13%, 60.64%, and 43.98%, respectively. Subsequent to treatment with high concentrations of ginsenosides, the viability of A549 cells dropped below 5%. Fluorescent images depicting cell viability validated these results, demonstrating a decrease in green fluorescence and an increase in red fluorescence with rising concentrations of ginsenosides in the serum-free group. Collectively, these results demonstrate that ginsenosides significantly inhibit the proliferation of A549 cells in a dose-dependent manner under serum starvation conditions. The structures of ginsenosides CK, Rh2(S), and Rg3(S), along with their IC_50_ values under serum-free conditions, are presented in Figure 2A. Based on the IC_50_ values, the concentrations used in subsequent experiments were set at 10 μM for ginsenosides CK and Rh2(S), and 50 μM for Rg3(S).

### 2.2. Ginsenoside Induces the Apoptosis of A549 Cells Under Serum Starvation

To further explore the impact of serum starvation on ginsenoside-induced apoptosis in A549 cells, the cells were treated with medium concentrations of ginsenosides for 24 h. Subsequently, cell morphology was characterized using Hoechst 33258 staining, as illustrated in Figure 2B. Apoptosis levels in A549 cells were evaluated using flow cytometry and subjected to quantitative analysis (refer to Figure 2C,D). The results indicate that under 10% serum culture conditions, no significant morphological changes were observed in either the treatment or control group, and there was no notable difference in apoptosis rates. However, under serum starvation conditions, a substantial increase in apoptosis was evident in the treatment group, characterized by decreased cell numbers and densely stained cell nuclei exhibiting blue-white fluorescence. Flow cytometry apoptosis analysis data supported these findings, showing a significant increase in apoptotic rates in the treatment group compared to the control group, particularly in early-stage apoptosis. Additionally, among the three types of ginsenosides, the ginsenoside CK exhibited the strongest induction of apoptosis in A549 cells, followed by Rh2(S). The interaction between serum starvation and ginsenoside treatment was analyzed using two-way ANOVA, and Figure S1A–C (see Supplementary Materials) indicate that the combined effect is synergistic.

### 2.3. Network Pharmacology Analysis of Ginsenosides

**Figure 3:**
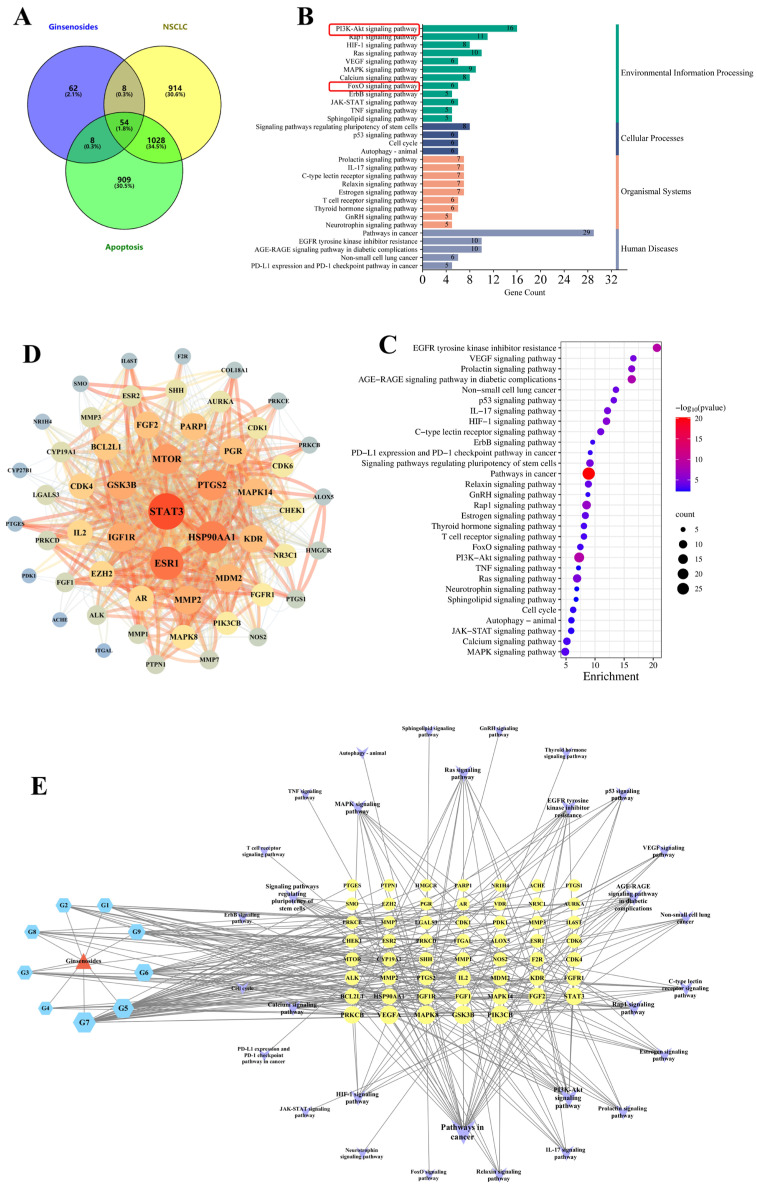
Results of network pharmacology analysis. (**A**) Diagram in Venn form displays the quantity of common targets among ginsenosides, NSCLC, and apoptosis. (**B**) PPI network of the common targets based on degree value assessed using Cytoscape software. (**C**) The top 30 most-enriched KEGG categories for the common targets show the vital ginsenoside-related signaling pathway against NSCLC according to network pharmacology analysis. (**D**) KEGG secondary classification of the top 30 signaling pathways. (**E**) Component–target–pathway network diagram; blue represents components, yellow represents targets, and purple represents pathways.

To elucidate the mechanism underlying ginsenoside-induced apoptosis in A549 cells under serum starvation conditions, we conducted a network pharmacology analysis. Previous investigations have revealed that ginsenosides CK, Rh2(S), and Rg3(S) undergo oxidative deglycosylation during hepatic metabolism, resulting in metabolites such as monooxygenated ginsenoside Rh2(S), protopanaxadiol-type saponin, and monooxygenated protopanaxadiol [15]. Therefore, we employed ginsenosides CK, Rh2(S), and Rg3(S) and their metabolites as drug components and predicted their targets using the SwissTargetPrediction platform. Detailed information regarding the drug components is presented in Table 1. Subsequently, the drug targets were intersected with targets associated with NSCLC and cell apoptosis, yielding 54 intersecting targets (Figure 3A). These targets were input into the STRING database, with species limited to Homo sapiens, resulting in a protein–protein interaction network. We conducted visualization analysis using Cytoscape (Figure 3B), revealing a network comprising 54 nodes and 880 edges. Employing the cytoHubba plugin, we identified the top three key targets ranked by degree as STAT3, ESR1, and HSP90AA1. KEGG pathway enrichment screening identified a total of 117 enriched terms, excluding nonspecific entries. The terms were sorted by *p*-value and the number of enriched targets, and we depicted the top 30 enriched terms as a bubble plot (Figure 3C). Among them, cancer pathways, the phosphoinositide 3-kinase (PI3K)/protein kinase B (Akt) signaling pathway, the Ras signaling pathway, and the Ras-related protein 1 (Rap1) signaling pathway emerged as potentially significant pathways. These pathways were categorized according to KEGG classification into four classes: environmental information processing, cellular processes, organismal systems, and human diseases (Figure 3D). Within the environmental information processing category, pathways related to nutrient metabolism were selected, revealing the PI3K/Akt signaling pathway and the forkhead box O (FoxO) signaling pathway. Previous studies have demonstrated that the FoxO signaling pathway is downstream of the PI3K/Akt signaling pathway and is regulated by it [28]. Thus, it is hypothesized that under serum starvation conditions, ginsenosides may induce apoptosis in A549 cells through the PI3K/Akt/FoxO signaling pathway. Furthermore, we constructed a visual network depicting the connectivity between ginsenosides, targets, and signaling pathways (Figure 3E). From the network, it is evident that ginsenosides act on multiple targets and pathways, particularly the PI3K/Akt signaling pathway, to promote cell apoptosis, thereby exerting therapeutic effects on NSCLC.

### 2.4. Bioinformatics Analysis of Ginsenosides

**Figure 4:**
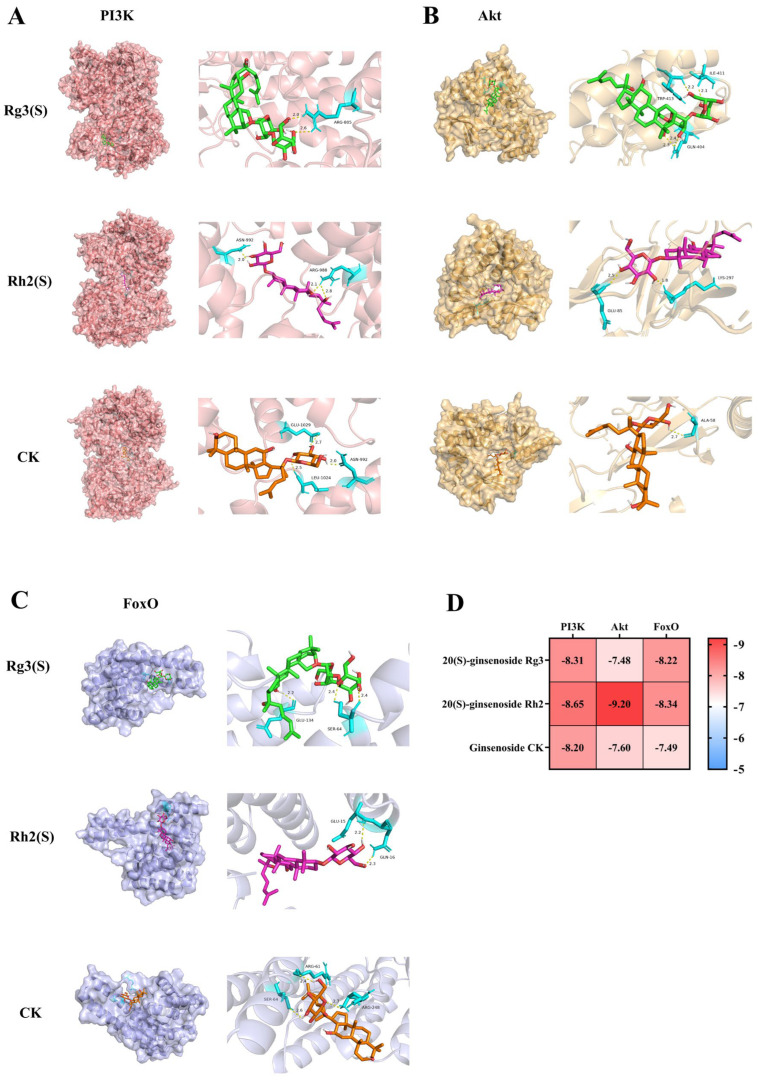
Results of molecular docking. Visualization of binding patterns of ginsenosides CK, Rh2(S), and Rg3(S) to PI3K (**A**), Akt (**B**), and FoxO (**C**), respectively. (**D**) Heatmap of binding energy between ginsenosides and target proteins.

The degree of receptor–ligand binding can be assessed by the level of binding energy as observed in molecular docking results. If the binding energy is less than −5.0 kcal·mol^−1^, it suggests a certain binding activity between the target and the compound, with lower binding energies indicating better docking efficacy. Molecular docking was performed between ginsenosides CK, Rh2(S), and Rg3(S), and the key targets PI3K, Akt, and FoxO within the PI3K/Akt/FoxO signaling pathway. Specific docking results are elaborated on in Table 2 and Figure 4D. Detailed docking conformations are illustrated in Figure 4A–C. Visual inspection of molecular docking results reveals that ginsenosides enter the active sites of the key target proteins and interact with certain amino acid residues through the formation of multiple hydrogen bonds. Furthermore, within the visualization results, the hydrogen bond lengths formed between ginsenosides and the key targets are all less than 3 Å, indicating close proximity between the amino acids and the compounds, thereby suggesting tight binding. The docking results indicate that ginsenosides CK, Rh2(S), and Rg3(S) form stable interactions with the key targets PI3K, Akt, and FoxO, suggesting that ginsenosides can spontaneously bind to target proteins, thereby inducing cell apoptosis and exerting therapeutic effects on NSCLC.

### 2.5. Ginsenoside Promotes the Apoptosis of A549 Cells by Regulating Key Molecules in the PI3K/Akt/FoxO Signaling Pathway Under Serum Starvation

**Figure 5:**
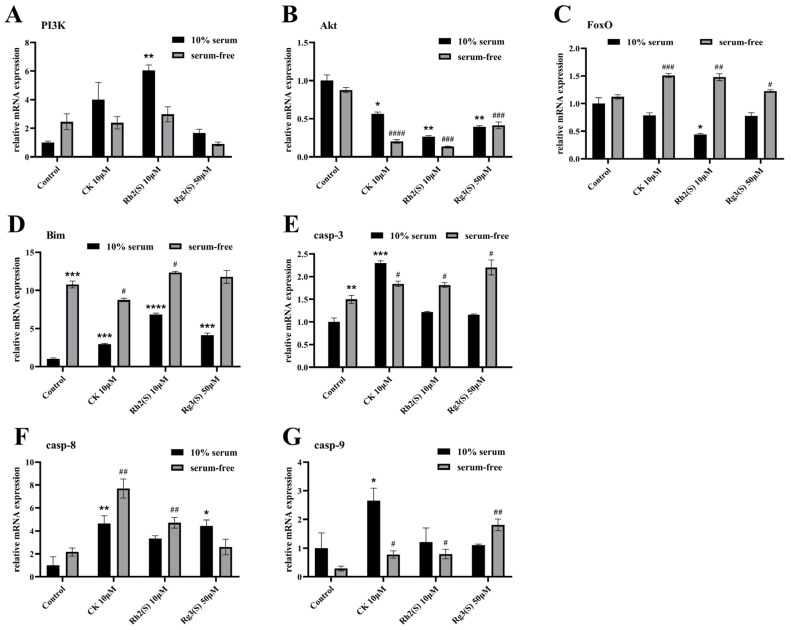
Ginsenosides induced mRNA transcript expression level changes in A549 cell lines in vitro. The expression levels of PI3K (**A**), Akt (**B**), FoxO (**C**), Bim (**D**), Caspase-3 (**E**), Caspase-8 (**F**), and Caspase-9 (**G**) were determined by qRT-PCR after ginsenoside administration for 24 h. Error bars represent mean ± SD; *n* = 3. (* *p* < 0.05, ** *p* < 0.01, *** *p* < 0.001, and **** *p* < 0.0001 vs. the control of the 10% serum group; # *p* < 0.05, ## *p* < 0.01, ### *p* < 0.001, and #### *p* < 0.0001 vs. the control of the serum-free group).

Validation of the bioinformatics analysis results of ginsenosides was conducted using quantitative reverse transcription-polymerase chain reaction (qRT-PCR) detection technology. A549 cells in both the 10% serum and serum-free groups were treated with moderate concentrations of ginsenosides for 24 h, followed by mRNA expression level measurements of key molecules in the signaling pathway, including PI3K, Akt, and FoxO (refer to Figure 5A–C). In the 10% serum group, the addition of ginsenosides resulted in an increase in PI3K mRNA expression levels and a decrease in Akt and FoxO mRNA expression levels. In the control samples of both the 10% serum and serum-free groups, there were no significant differences in the mRNA expression levels of the three key molecules, indicating that serum starvation alone did not affect the PI3K/Akt/FoxO signaling pathway. However, under serum starvation conditions with ginsenoside treatment, the mRNA expression level of Akt in A549 cells significantly decreased, to even lower than that observed with ginsenoside treatment alone. Notably, the mRNA expression level of FoxO significantly increased, contrary to the results of ginsenoside treatment alone. Overall, under serum starvation conditions, ginsenosides inhibit the expression of Akt and activate FoxO in serum-starved A549 cells.

Concurrently, changes in the mRNA expression levels of apoptosis-related proteins were examined, including the pro-apoptotic molecules Bcl-2-like protein 11 (Bim), and Caspase family proteins such as Caspase-3, Caspase-8, and Caspase-9 downstream of the PI3K/Akt/FoxO signaling pathway (Figure 5D–G). The results revealed that in A549 cells treated solely with ginsenosides, the mRNA expression levels of the aforementioned pro-apoptotic proteins increased. Treatment solely with serum starvation led to increased mRNA expression levels of both Bim and Caspase-3. Under serum starvation conditions with ginsenoside treatment, significant increases in the expression levels of Bim, Caspase-3, Caspase-8, and Caspase-9 were observed. Particularly noteworthy was the increase in Caspase-8, whose mRNA expression level exceeded that of treatment with ginsenosides alone. This indicates that under conditions of serum starvation, ginsenosides primarily induce apoptosis in A549 cells by increasing the mRNA expression levels of Caspase-8. This outcome is likely mediated by the inhibition of Akt expression by ginsenosides, thereby promoting FoxO expression.

### 2.6. Ginsenosides Decreased the Mitochondrial Membrane Potential of A549 Cells Under Serum Starvation

**Figure 6:**
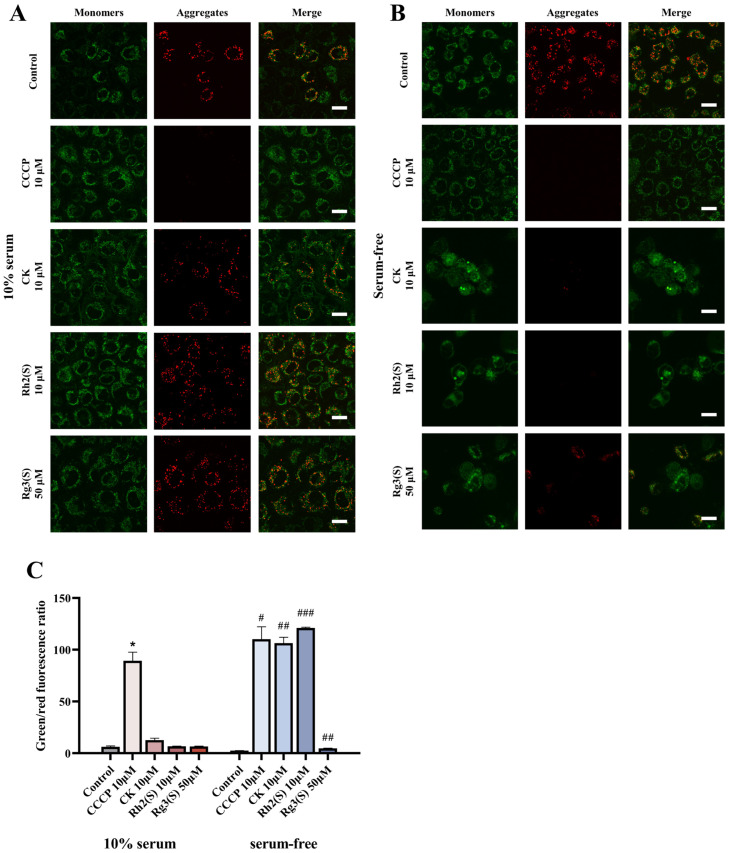
Ginsenosides induce the loss of mitochondrial membrane potential (ΔΨm) in A549 cells under serum starvation. (**A**) JC-1 staining in cells cultured with 10% serum. Treatment with ginsenosides alone did not alter ΔΨm, as indicated by predominant red fluorescence. (**B**) JC-1 staining under serum-free conditions. Serum starvation alone did not reduce ΔΨm, while combined treatment with ginsenosides strongly enhanced green fluorescence, indicating ΔΨm loss. (**C**) Quantitative analysis of the JC-1 monomer/aggregate ratio. A significant increase in the ratio was observed only in serum-starved cells treated with ginsenosides (# *p* < 0.05, ## *p* < 0.01, and ### *p* < 0.001 vs. serum-free control; * *p* < 0.05 vs. 10% serum control). Scale bar: 20 μm. Error bars represent mean ± SD; *n* = 3.

Cell apoptosis typically involves a decrease in mitochondrial membrane permeability [29]. We assessed the mitochondrial membrane potential of A549 cells using JC-1 staining. When mitochondria have a high membrane potential, JC-1 forms aggregates emitting red fluorescence, whereas a low membrane potential results in green fluorescence. Figure 6A,B depict fluorescence micrographs of cellular mitochondrial membrane potential, and Figure 6C presents the corresponding fluorescence quantification. The ratio of green to red fluorescence reflects changes in mitochondrial membrane potential; higher ratios indicate lower membrane potentials. In Figure 6A, cells treated with various concentrations of ginsenosides exhibited red fluorescence, and their mitochondrial membrane potentials did not significantly differ from those of the control group, indicating that treatment with moderate concentrations of ginsenosides alone does not reduce mitochondrial membrane potential. Similarly, the control group in Figure 6B also displayed noticeable red fluorescence, suggesting that serum starvation alone does not alter mitochondrial membrane potential. However, under serum starvation conditions with ginsenoside treatment, a significant reduction in mitochondrial membrane potential was observed in A549 cells, accompanied by a sharp decrease in cell count and enhanced green fluorescence, with weakened or absent red fluorescence in the fluorescence micrographs. Notably, the results for ginsenosides CK and Rh2(S) closely resembled those of the positive control carbonyl cyanide 3-chlorophenylhydrazone (CCCP), suggesting that, under serum starvation, ginsenosides can decrease the mitochondrial membrane potential of A549 cells.

NSCLC represents the most prevalent form of lung cancer, characterized by reported five-year survival rates of merely 16% in advanced stages. Researchers worldwide have devoted substantial efforts to pursue novel, highly efficient, and low-toxicity anti-NSCLC drugs [30,31]. Ginsenosides, the primary active components of ginseng, have garnered extensive attention and validation for their potential in anti-tumor therapy in recent years. Specifically, the protopanaxadiol-type ginsenosides Rg3(S), Rh2(S), and CK have exhibited notable anti-tumor activity and less biotoxicity, and are frequently employed in conjunction with chemotherapy drugs for clinical lung cancer management. Based on the current literature, under 10% serum culture conditions, the 24 h IC50 values of Rh2(S) and CK against lung cancer A549 cells were 59.55 μM and 40.83 μM, respectively, whereas the IC50 value of Rg3(S) exceeded 50 μM [32,33,34,35]. Significantly, under serum starvation conditions, the 24 h IC50 values of all three ginsenosides against A549 cells were notably reduced, with Rh2(S) and CK even below 10 μM. Cell viability assays revealed that under serum starvation conditions, ginsenosides had a stronger inhibitory effect on the proliferation of A549 cells. Apoptosis assays indicated that under serum starvation conditions, ginsenosides elicited early apoptosis in A549 cells. Similar findings were confirmed in studies employing Rh2(S) and Rg3(S) for cervical cancer treatment [36,37].

The KEGG pathway enrichment analysis indicated that the targets were predominantly enriched in the PI3K/Akt/FoxO signaling pathway. qRT-PCR experiments revealed decreased mRNA expression of Akt and increased mRNA expression of FoxO under serum starvation conditions induced by ginsenosides. The PI3K/Akt/FoxO signaling pathway, as a central pathway governing cell growth and metabolism, plays a role in modulating various physiological processes, such as cell proliferation, apoptosis, and insulin resistance [38,39]. In particular, the PI3K/Akt pathway negatively regulates FoxO transcriptional activity, leading to the expression of the pro-apoptotic protein Bim, thereby facilitating epithelial cell apoptosis [40,41]. Moreover, the FoxO family of transcription factors might influence mitochondrial membrane potential by regulating the expression of mitochondria-related genes, thereby activating both mitochondrial-dependent and -independent apoptosis pathways [42,43]. Under serum starvation conditions, a reduction in mitochondrial membrane potential was observed alongside the increased mRNA expression of the pro-apoptotic proteins Bim, Caspase-3, Caspase-8, and Caspase-9, suggesting that ginsenosides could induce typical apoptosis through both extrinsic and intrinsic pathways.

**Figure 7:**
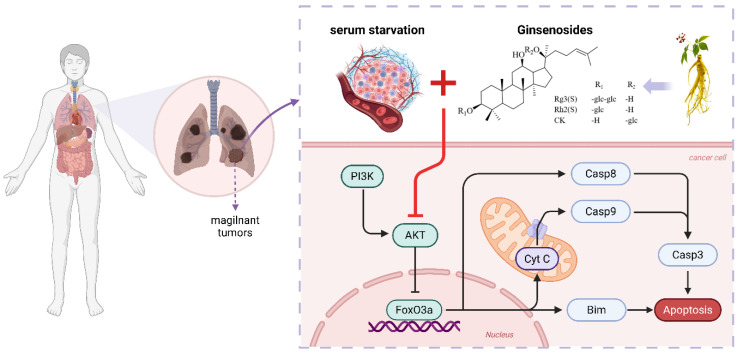
Schematic diagram of the mechanism through which ginsenosides inhibit the proliferation of and promote apoptosis in A549 cells via the PI3K/Akt/FoxO pathway under serum starvation.

This study hypothesizes that, under serum starvation conditions, ginsenosides inhibit Akt and activate FoxO by regulating the PI3K/Akt/FoxO signaling pathway in A549 cells. The activation of FoxO decreases mitochondrial membrane potential, indirectly influencing the activity of the Caspase protein family. In the intrinsic pathway of apoptosis, the heightened permeability of the mitochondrial membrane results in the release of specific proteins (such as cytochrome c) into the cytoplasm, subsequently activating the Caspase protein family and initiating apoptosis [44]. Figure 7 illustrates the potential mechanistic pathway through which serum starvation may enhance ginsenoside-induced apoptosis in A549 lung cancer cells.

Indeed, when compared to cell cultures under normal serum conditions, cells subjected to serum starvation display a survival state more closely resembling the actual tumor microenvironment in vivo. The network pharmacology analysis results revealed that G7, G5, and G6 were the top three active ingredients of ginsenosides, all of which are metabolic products of ginsenosides Rg3(S), Rh2(S), and CK in vivo. Our prior studies have demonstrated that these metabolites exhibit stronger anti-tumor activities compared to the parent compounds [15]. The present investigation reveals that when cancer cells were placed in a nutrient-deficient environment, ginsenosides exhibited a stronger anti-tumor effect. As a result, the development of novel drug delivery systems to ensure the targeted accumulation of ginsenosides at tumor sites post-administration represents a promising strategy for enhancing their therapeutic efficacy.

The strong docking scores obtained in this study suggest promising interactions between the compounds and the target of interest. It should be noted, however, that molecular docking is a predictive computational tool and cannot independently confirm actual binding or functional activity in a biological context. While these results provide a supportive in silico model, future experimental validation using techniques such as surface plasmon resonance (SPR) or isothermal titration calorimetry (ITC) to quantify binding affinity, together with functional cellular assays, will be essential to unequivocally establish the biological significance of these interactions.

## 3. Materials and Methods

### 3.1. Materials

Ginsenosides CK, Rh2(S), and Rg3(S) (purity > 98%) were purchased from Chengdu Must Bio-Technology (Chengdu, China). A549 cells were obtained from the Shanghai Cell Bank of the Chinese Academy of Science (Shanghai, China). RPMI-1640 medium and penicillin-streptomycin were ordered from Hyclone (Logan, UT, USA). Fetal bovine serum (FBS) was ordered from Sorfa (Huzhou, China). Also, 0.25% trypsin−EDTA solution was ordered from Gibco (San Francisco, CA, USA). Dimethyl sulfoxide (DMSO) and RNAsimple total RNA kit were ordered from Solarbio (Beijing, China). The cell counting kit-8 (CCK-8) assay was ordered from APExBIO (Shanghai, China). The calcein/propidium iodide (PI) cell viability/cytotoxicity assay kit, Hoechst 33258 staining kit, Annexin V-FITC apoptosis detection kit, and mitochondrial membrane potential assay kit with J-aggregates (JC-1) were purchased from Beyotime (Shanghai, China). The FastKing RT kit and SYBR Premix Ex Taq were purchased from Tiangen (Beijing, China).

### 3.2. Culture of A549 Cells

A549 lung cancer cells were cultured in RPMI-1640 medium supplemented with 10% FBS and 1% penicillin-streptomycin as a complete growth medium. Cells were incubated in a CO_2_ incubator maintained at 37 °C with 5% CO_2_. Cells were divided into two groups for treatment: Group 1 received a complete medium supplemented with 10% FBS (10% serum), and Group 2 underwent serum starvation with 0% FBS (serum-free).

### 3.3. Cell Viability Assay

A549 cells were treated with various concentrations of ginsenoside for 24 h. Cell viability was assessed using the cell counting kit-8 (CCK-8) assay and calcein/propidium iodide (PI) cell viability/cytotoxicity assay according to the manufacturer’s protocol. The results of the CCK-8 assay were determined by measuring the optical density at 450 nm in each well using a microplate reader (Bio-Rad, Hercules, CA, USA). After incubation with the calcein/PI buffer for 30 min, live and dead cells were observed under a fluorescent microscope.

### 3.4. Hoechst 33258 Staining Detection

The detection of apoptosis in A549 cells was performed using Hoechst 33258 staining. A549 cells were incubated with various concentrations of ginsenoside for 24 h, followed by fixation with 4% formaldehyde for 15 min at room temperature, and then stained with Hoechst 33258 for 15 min. Cells with nuclei containing condensed chromatin or fragmented nuclei were identified as apoptotic cells through visualization.

### 3.5. Flow Cytometry Assays

After 24 h of treatment with ginsenosides, A549 cells were stained with the Annexin V-FITC apoptosis detection kit. Flow cytometry (CytoFLEX, Beckman, CA, USA) was employed to analyze the samples and quantify the percentage of apoptotic cells.

### 3.6. Bioinformatics Analysis

The bioinformatics analysis was conducted according to the protocol outlined by Aqsa Kanwal and colleagues [45]. Chemical structures were prepared with ChemDraw software (version 20.0.0.41), and the corresponding canonical SMILES were analyzed using the SwissTargetPrediction platform to predict potential targets (http://www.swisstargetprediction.ch/) (accessed on 8 October 2024). The predicted targets were identified and validated through the UniProt database (https://www.uniprot.org/) (accessed on 8 October 2024). Subsequent network pharmacology analysis was performed using Cytoscape software (version 3.9.1).

Network pharmacology was utilized to preliminarily predict potential targets, pathways, and mechanisms associated with the anti-NSCLC activity of bioactive components. The GeneCards and DisGeNET databases were employed to identify NSCLC-related genes using the keyword “non-small cell lung cancer,” and cell apoptosis-related genes were selected using the keyword “apoptosis” [46,47]. Common targets of ginsenosides against NSCLC were identified based on their induction of cell apoptosis, illustrated in a Venn diagram generated using the Venn website [48]. Additionally, a protein–protein interaction (PPI) network model, with the species set to “Homo sapiens,” was constructed using the STRING database online platform [49]. Kyoto Encyclopedia of Genes and Genomes (KEGG) pathway analysis was performed using the Database for Annotation, Visualization, and Integrated Discovery (DAVID), and network relationships were visualized using Cytoscape software [50].

Protein–ligand docking was performed using AutoDock Vina (version 1.1.2) to predict the predominant binding mode of the ligand with the protein [51]. The active constituents were employed as ligands, whereas the targets were utilized as receptors. Subsequently, the optimal docking results were chosen and visualized with PyMOL (version 2.6.0a0).

### 3.7. Quantitative Reverse Transcription-Polymerase Chain Reaction (qRT-PCR)

The qRT-PCR analysis utilized the CFX96™ real-time system (Bio-Rad, USA). A 20 µL reaction mixture was prepared, comprising 2 µL of template, 0.6 µL of each primer (400 nM final concentration), and 10 µL of 2 × Talent qPCR PreMix (Tiangen, Beijing, China). The PCR protocol followed the manufacturer’s guidelines, starting with an initial denaturation at 95 °C for 3 min, followed by 40 cycles of 5 s at 95 °C and 15 s at 60 °C. Melting curve analysis included 30 s at 95 °C, 30 s at 65 °C, and 30 s at 95 °C. Normalization against β-actin mRNA levels enabled relative gene expression quantification using the 2-ΔΔCT method. The primer sequences are provided in Table 3.

### 3.8. Mitochondrial Membrane Potential Measurement

Mitochondrial membrane potential (MMP) in cells was assessed using a mitochondrial membrane potential detection kit (JC-1) (C2006, Beyotime, Shanghai, China). A549 cells were washed and then incubated with JC-1 working solutions for 30 min at 37 degrees Celsius in the incubator. Additionally, those treated with carbonyl cyanide 3-chlorophenylhydrazone (CCCP) served as negative controls during MMP evaluation, following the provided instructions. Subsequently, live cells were imaged using a confocal laser scanning microscope (FV3000, Olympus, Tokyo, Japan) after incubation. Confocal images were rapidly acquired and then quantified for mean fluorescence intensities in arbitrary regions using ImageJ (version 1.52a).

### 3.9. Statistics and Analysis

All experiments in this study were conducted with at least three independent replicates, and data are presented as the mean ± standard deviation. The normality (Shapiro–Wilk test) and homoscedasticity (Levene’s test) of data were confirmed prior to analysis. Statistical significance between treatment groups was determined using one-way ANOVA followed by Tukey’s post hoc test for multiple comparisons. Significance levels are denoted as follows: * *p* < 0.05, ** *p* < 0.01, *** *p* < 0.001, and **** *p* < 0.0001.

## 4. Conclusions

In summary, we assessed the anti-lung cancer activity of ginsenosides under serum starvation conditions and explored their potential mechanisms. Our results demonstrated that ginsenosides inhibited the proliferation of and induced apoptosis in A549 cells under serum deprivation, suggesting potent anti-tumor effects. Mechanistically, our multi-faceted approach indicated that ginsenosides likely regulate the PI3K/Akt/FoxO signaling pathway, as evidenced by inhibited Akt and activated FoxO expression. Nevertheless, a limitation of this study is its reliance on mRNA expression and bioinformatic predictions to infer pathway involvement. Future studies employing pathway-specific inhibitors or genetic approaches are essential to conclusively establish causality. By simulating the nutrient-deprived tumor microenvironment in vitro, we provided evidence that ginsenosides may exert enhanced anti-tumor effects under metabolic stress. These findings offer valuable insights for developing ginsenosides as potential clinical therapeutics targeting adverse tumor microenvironments.

## Figures and Tables

**Table 1:** Information on active ingredients of ginsenosides.

Number	PubChem CID	Molecule Name	Degree
G1	9918693	20(S)-Ginsenoside Rg3	10
G2	119307	20(S)-ginsenoside Rh2	10
G3	9852086	Ginsenoside CK	9
G4	118753486	20(S)-Ginsenoside Rh2 Metabolite M1-1	7
G5	11213350	20(S)-Protopanaxadiol	22
G6	101228398	Protopanaxadiol Oxide	17
G7	118753219	20(S)-Protopanaxadiol Metabolite M1-1	25
G8	12314836	20(S)-Protopanaxadiol Metabolite M1-2	9
G9	73352321	20(S)-Protopanaxadiol Metabolite M1-3	13

**Table 2:** Ginsenosides docking with key proteins.

Target Protein	PDB ID	Compound	Affinity (kcal/mol)	H Bonds
PI3K	4BFR	Rg3(S)	−8.312	ARG805 (2.0 Å, 2.6 Å)
		Rh2(S)	−8.647	ARG988 (2.1 Å, 2.8 Å), ASN992 (2.0 Å)
		CK	−8.201	ASN992 (2.0 Å), GLU1029 (2.7 Å), and LEU1024 (2.5 Å)
Akt	6HHI	Rg3(S)	−7.475	GLN404 (2.3 Å, 2.4 Å), ILE411 (2.1 Å), and TRP413 (2.2 Å)
		Rh2(S)	−9.203	GLU85 (2.5 Å), LYS297 (1.8 Å)
		CK	−7.602	ALA58 (2.7 Å)
FoxO	7V9B	Rg3(S)	−8.223	GLU134 (2.2 Å), SER64 (2.4 Å, 2.4 Å)
		Rh2(S)	−8.342	GLN16 (2.3 Å), GLU15 (2.2 Å)
		CK	−7.486	ARG61 (2.4 Å), ARG248 (2.7 Å), and SER64 (2.6 Å)

**Table 3:** Primers used in qRT-PCR.

Gene	Forward Primers (From 5′ to 3′)	Reverse Primers (From 5′ to 3′)
PI3K	GGTTGGTGGCTGTTCTTACTGTC	CAAGTCTGGCTGGAATGATGCTAT
AKT	CCACTGTCATCGAACGCACCTT	GAAGTCCATCTCCTCCTCCTCCTG
FoxO3a	AGTTCCCTCATTCTGGACCC	CTTCAAGGATAAGGGCGACA
Bim	GATAGTGGTTGAAGGCCTGG	CCTCCCTACAGACAGAGCCA
Caspase-3	TGCAGTCATTATGAGAGGCAAT	AAGGTTTGAGCCTTTGACCA
Caspase-8	GAAGATAATCAACGACTATG	TTCACTATCCTGTTCTCT
Caspase-9	ACATGCTGGCTTCGTTTCTG	TCTCAAGAGCACCGACATCA
β-actin	GCAAGCAGGAGTATGACGAG	CAAATAAAGCCATGCCAATC

## Data Availability

Data are contained within the article.

## Supplementary Materials

The following supporting information can be downloaded at: https://www.mdpi.com/article/10.3390/molecules30183697/s1, Figure S1: Synergistic effects of ginsenosides and serum starvation on apoptosis in A549 cells. Interaction effects between (A) ginsenoside CK, (B) ginsenoside Rh2(S), (C) ginsenoside Rg3(S) and serum starvation were analyzed by two-way ANOVA. Results indicate a statistically significant synergistic interaction for all three ginsenosides (*p* < 0.05), suggesting enhanced apoptotic effects under serum-deprived conditions. Error bars represent mean ± SD; *n* = 3. (* *p* < 0.05, ** *p* < 0.01, *** *p* < 0.001).

## Abbreviations

The following abbreviations are used in this manuscript:

Akt	Protein kinase B
Bim	Bcl-2-like protein 11
CDK4	Cyclin-dependent kinase 4
FoxO	Forkhead box O
HES1	Hairy and enhancer of split 1
ITC	Isothermal titration calorimetry
JNK	c-Jun N-terminal kinase
MAPKs	Mitogen-activated protein kinases
NSCLC	Non-small-cell lung cancer
Notch	Neurogenic locus notch homolog protein
PI3K	Phosphoinositide 3-kinase
p53	Tumor protein 53
