# Supplementary material for: Ginsenosides Enhanced Apoptosis of Serum-Free Starved A549 Lung Cancer Cells
Source: Molecules. 2025 Sep 11;30(18):3697. doi: 10.3390/molecules30183697 (PMC12472411; doi:10.3390/molecules30183697)
Supplement: Supplementary file 1 [file molecules-30-03697-s001.zip › molecules-3829272-supplementary.pdf]

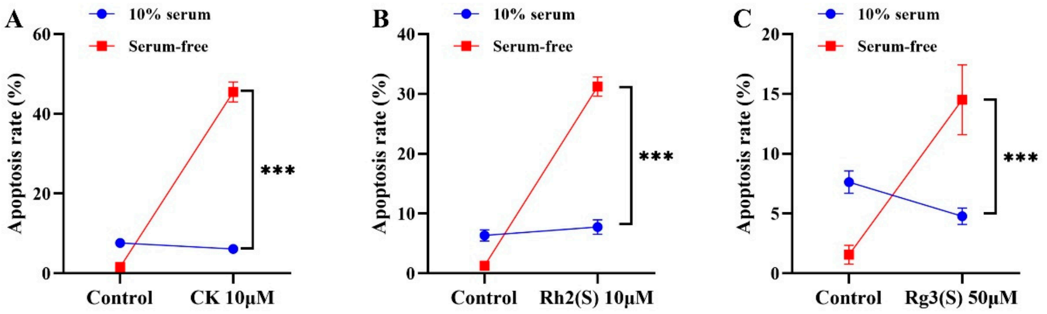

Figure S1. Synergistic effects of ginsenosides and serum starvation on apoptosis in A549 cells. Interaction effects between **(A)** ginsenoside CK, **(B)** ginsenoside Rh2(S), **(C)** ginsenoside Rg3(S) and serum starvation were analyzed by two-way ANOVA. Results indicate a statistically significant synergistic interaction for all three ginsenosides ($p < 0.05$), suggesting enhanced apoptotic effects under serum-deprived conditions. Error bars represent mean ± SD; n = 3. (*P < 0.05, **P < 0.01, ***P < 0.001)
